# Modulation of the NF-κB signaling pathway by the combined strategy of tocilizumab and dexamethasone for asthma therapy

**DOI:** 10.1186/s12931-025-03458-5

**Published:** 2026-01-08

**Authors:** Xinyi Li, Jingyi Zhang, Yihui Feng, Qihui Zhou, Chunling Zhang

**Affiliations:** 1 Qingdao Medical College, Qingdao University, Qingdao, 266073 China; 2 Department of Pulmonary and Critical Care Medicine, Qingdao Central Hospital, NHC Key Laboratory of Cardiopulmonary Rehabilitation and Functional Recovery (University of Health and Rehabilitation Sciences), Shandong Engineering Research Center for Tissue Rehabilitation Materials and Devices, School of Rehabilitation Sciences and Engineering, University of Health and Rehabilitation Sciences, Qingdao, 266113 China

**Keywords:** Asthma, Inflammation, Tocilizumab, Dexamethasone, NF-κB signaling pathway

## Abstract

Asthma, a prevalent chronic inflammatory airway disease, is conventionally managed with glucocorticoids (GCs) such as dexamethasone (DEX). However, prolonged DEX use induces adverse effects, including immunosuppression and metabolic disruptions. Tocilizumab (TCZ), a humanized monoclonal antibody with immunomodulatory and anti-inflammatory properties, may enhance therapeutic efficacy while minimizing DEX-related toxicities. Herein, we evaluate a combined therapeutic strategy integrating TCZ with a reduced DEX dose (TCZ + DEX) for asthma management. In lung epithelial cells subjected to hydrogen peroxide-induced oxidative stress, TCZ + DEX improved cell viability, reduced apoptosis, and supported proliferative activity, thereby attenuating oxidative and inflammatory injury. The combination therapy also decreased reactive oxygen species levels, preserved mitochondrial membrane potential, and modulated cell cycle-related gene expression, suggesting protection against oxidative stress–induced bronchial epithelial injury and reversal of DEX-mediated cell cycle arrest. In an ovalbumin-induced asthma model, TCZ + DEX improved lung function, reduced airway inflammation, and attenuated airway remodeling, achieving efficacy comparable to standard-dose DEX despite reduced steroid exposure. Mechanistically, TCZ + DEX was associated with suppression of the nuclear factor kappa B–related inflammatory signaling, thereby attenuating proinflammatory cytokine production. These findings provide preclinical evidence that TCZ + DEX offers a practical and safer therapeutic strategy for asthma, mitigating GC-associated adverse effects and providing insights into novel approaches for other chronic inflammatory diseases.

**Supplementary Information:**

The online version contains supplementary material available at 10.1186/s12931-025-03458-5.

## Introduction

Asthma, a complex chronic inflammatory disease of the airways affecting over 300 million individuals globally, is regulated by multifaceted mechanisms involving genetic, environmental, and immune system factors [1, 2]. It is characterized by persistent airway inflammation, increased bronchial sensitivity, and airway structural alterations, with chronic inflammation serving as the central initiator and regulator of disease progression [3]. For example, Th2 cell activation is critically involved in sustaining chronic inflammation by secreting cytokines such as interleukin-4 (IL-4), interleukin-5 (IL-5), and interleukin-13 (IL-13), which drive eosinophil infiltration, immunoglobulin E (IgE) mediated allergic responses, and increased mucus production [4]. These processes not only induce excessive contraction of airway smooth muscles and airway narrowing (airway hyperresponsiveness) but also contribute to abnormal airway remodeling through epithelial damage and basement membrane thickening [3], ultimately leading to airflow limitation and the clinical manifestations of breathlessness. Current asthma treatment primarily relies on traditional anti-inflammatory drugs and bronchodilators, such as corticosteroids (e.g., dexamethasone) and β2-adrenergic agonists [5]. Dexamethasone (DEX) is clinically administered to alleviate asthma symptoms through suppressing inflammatory responses, inhibiting immune cell activity, and reducing airway remodeling. However, prolonged or high-dose use of DEX causes adverse effects such as osteoporosis, diabetes, slowed growth, respiratory infections, and Cushing’s syndrome [6, 7]. To mitigate these side effects, a combined approach utilizing DEX with other anti-inflammatory agents is considered an effective strategy to reduce corticosteroid dependency [8].

Tocilizumab (TCZ) is a humanized monoclonal antibody that specifically targets the human interleukin-6 receptor (IL-6R) and is used to treat various immune-mediated diseases [9]. Previous studies have confirmed its efficacy in reducing acute-phase responses and lowering levels of acute-phase proteins, such as C-reactive protein, thereby suppressing systemic inflammation [10]. Specifically, TCZ exerts significant anti-inflammatory effects by modulating the activation of pro-inflammatory pathways and reducing the production of pro-inflammatory cytokines such as tumor necrosis factor alpha (TNF-α) [11]. Additionally, it regulates abnormal immune responses in T and B cells, restores immune system balance, and improves the pathological conditions of autoimmune diseases [12, 13]. TCZ has been established as a treatment for rheumatoid arthritis, systemic juvenile idiopathic arthritis, and COVID-19 [14, 15]. Although TCZ has not yet been approved for clinical use in asthma, previous studies have demonstrated that interleukin-6 (IL-6) levels are significantly elevated in the bronchoalveolar lavage fluid (BALF) of patients with asthma, suggesting its involvement in airway inflammation [16]. These findings indicate potential advantages of combining TCZ with DEX for asthma treatment, which warrants further investigation.

**Fig. 1:**
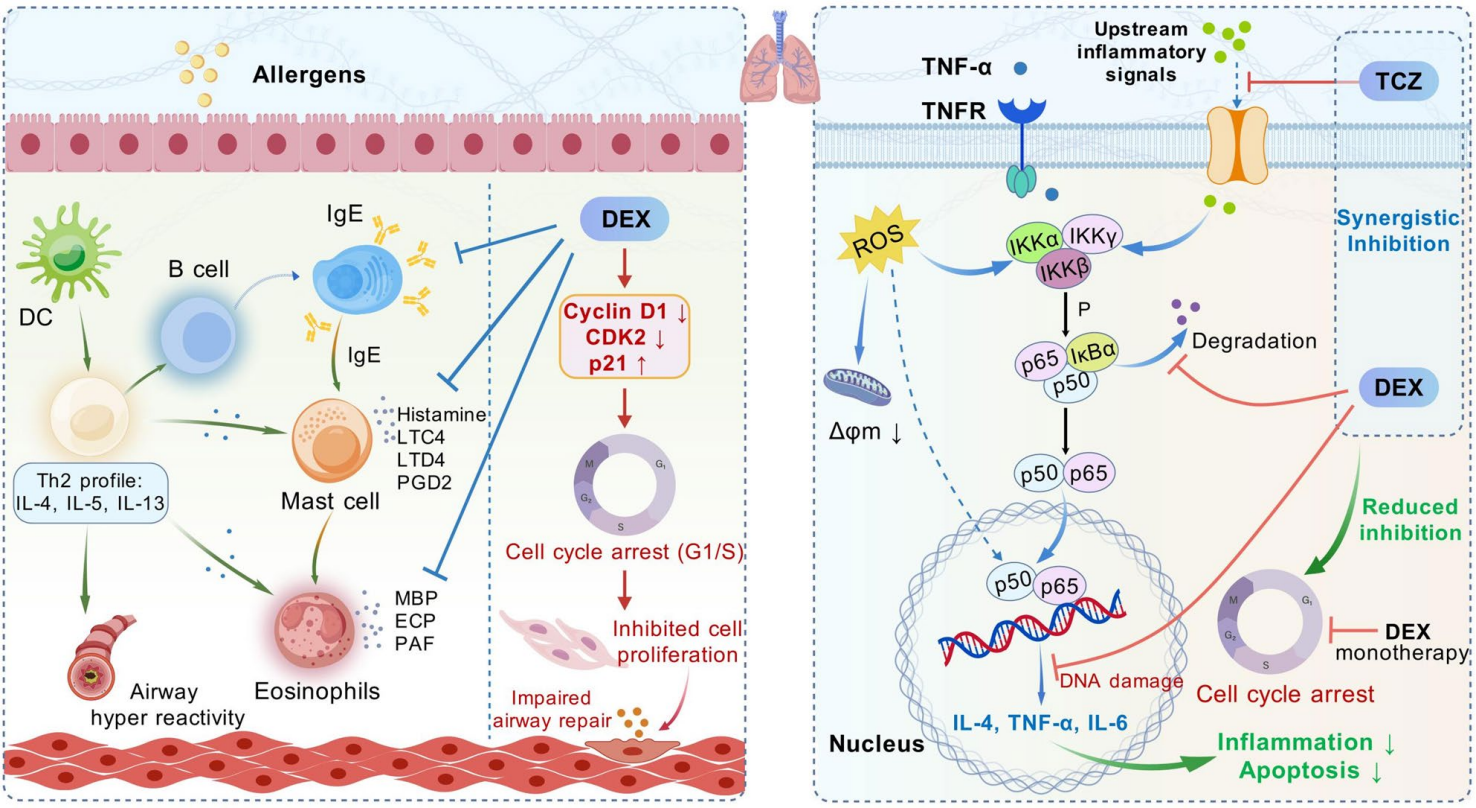
TCZ combined with DEX activates the NF-κB pathway and alleviates the inhibitory influence exerted DEX on the cell cycle. Created with BioGDP.com and obtained its permission

Herein, this study aims to investigate the therapeutic efficacy of combining TCZ with DEX in treating asthma, with the goal of reducing DEX dependency and mitigating adverse effects associated with high-dose steroid use in clinical administration (Fig. 1). In vitro, the combined therapy was evaluated for it*s* ability to alleviate inflammation-related cell damage in human bronchial epithelial cells (BEAS-2B), which were subjected to oxidative stress *via* hydrogen peroxide (H_2_O_2_) treatment. Afterwards, Cell Counting Kit-8 (CCK-8), live/dead cell staining, flow cytometry, reactive oxygen species (ROS) detection, JC-1 staining, and quantitative real-time PCR (RT-qPCR) were conducted to assess cellular survival, proliferative capacity, and apoptotic processes. In vivo, an asthma mouse model was established to assess the effects of the combination therapy on airway inflammation, pulmonary pathology, and lung function. Analytical methods included behavioral scoring, pulmonary function tests, enzyme-linked immunosorbent assay (ELISA) for cytokines in serum and BALF, histopathological examination, immunohistochemistry, RT-qPCR, and Western blotting. This comprehensive research aims to elucidate the combined therapeutic effects and underlying mechanisms of combined TCZ and DEX administration, offering new theoretical insights into their potential as a therapeutic strategy for asthma.

## Materials and methods

### Effect of TCZ + DEX on mitigating the toxic side effects of inflammation on cells in vitro

#### Cell culture

BEAS-2B, obtained from FuHeng Biology, China, were cultured in Dulbecco’s Modified Eagle Medium (DMEM, Corning, #10–013-CV) supplemented with 10% fetal bovine serum (FBS, Pricella, #164210-50) and 1% penicillin/streptomycin (Solarbio, #P1400), and incubated at 37℃ with 5% CO_2_. Subculturing was performed at a 1:3 dilution once cell confluence reached approximately 85%.

#### Cell seeding

The cells were seeded at a density of 6 × 10^3^ cells/well in 96-well plates or 2 × 10^5^ cells/well in 6-well plates and then incubated at 37 °C for 24 h. Afterwards, 600 µM H_2_O_2_ (HuaSheng, Tianjin) was introduced to co-incubate the cells for an additional 24 h. Cells were then treated with 0.7 µM TCZ (Roche, Switzerland), 1 µM DEX (Solarbio, #D8040), or a combination of both drugs (terms as TCZ + DEX) containing 0.7 µM TCZ and 0.5 µM DEX, for 24 h. The cells were then prepared for the next experiment.

#### Observation and analysis of cell morphology

After the experiment, cells from each group were washed with PBS and observed under an inverted microscope (ZEISS, #Axio Vert A1) to assess cell morphology. Images were captured using a 20× objective lens, ensuring that five random fields of view were recorded for each group.

#### Analysis of cell proliferation, viability, and apoptosis

To assess cell proliferation, BEAS-2B cells seeded in 96-well plates were treated with the drug the next day. Then, the cell activity was assessed by CCK-8 at 6 h, 12 h, 24 h and 48 h after cell drug exposure. A 10 µL aliquot of CCK-8 reagent (Beyotime Biotechnology, #C0042) was transferred into wells, followed by 2 h incubation in culture medium. Subsequently, the absorbance at 450 nm was measured using a microplate reader (HEALES, #MB-530).

Cell viability was assessed using the Calcein-AM/PI Staining Kit (Meilunbio, #MA0361). In brief, cells seeded in 6-well plates were treated with drugs for 24 h. Then, 300 µL of staining working solution containing 2 µM Calcein-AM and 8 µM PI was added, and the samples were incubated in darkness at 37 °C for 30 min. The cells were ultimately observed under a fluorescence microscope (Olympus, #BX53). The percentage of viable cells and the viability ratio were analyzed with ImageJ software.

To analyze the cell apoptosis, drug treatments were administered to cells cultured in 6-well plates for 24 h and subsequently assessed by flow cytometry using the Annexin V-FITC Apoptosis Detection Kit (Solarbio, #CA1020). Briefly, the cells were rinsed with PBS three times, digested with ethylenediaminetetraacetic acid (EDTA)-free trypsin (MeilunBio, #MA0234), collected, and centrifuged. After three additional washes with PBS, the cells were re-suspended in 0.5 mL of annexin binding buffer (Solarbio, #CA1020), then incubated with Annexin V-FITC/PI for apoptosis detection *via* flow cytometry (Mindray, #BriCyte^®^ MX).

#### Antioxidant capacity

To detect intracellular ROS levels, 2′,7′-Dichlorodihydrofluorescein diacetate (DCFH-DA, Beyotime Biotechnology, #S0033S) was used as the fluorescent probe. A total of 2 × 10^5^ cells per well were seeded into 6-well plates. Following 24 h of drug exposure, the cells were collected by centrifugation (1000 rpm, 5 min), resuspended in medium containing 10 µM DCFH-DA, and incubated at 37℃ for 20 min. Imaging was performed using a fluorescence microscope (Olympus, #BX53). Finally, the fluorescence intensity was quantified by calculating the ratio of total fluorescence intensity to the number of cells.

The JC-1 assay kit (Beyotime Biotechnology, #C2003S) was used to measure Mitochondrial membrane potential (ΔΨm). A total of 2 × 10^5^ cells per well were plated into 6-well plates. Following 24 h of drug exposure, cells were rinsed with PBS and then incubated with 500 µL dyeing solution for 20 min. Afterward, they were washed twice using the staining buffer. Fluorescence was observed using a fluorescence microscope (Olympus, #BX53). The total fluorescence intensity was then normalized to the cell count for quantification.

#### Analysis of gene expression by RT-qPCR to evaluate cellular responses

To evaluate TCZ + DEX’s effect on inflammatory microenvironment, qPCR assay was performed to detect inflammatory response (such as *IL6* and *TNF*), Nuclear Factor kappa B *(NF-κB)* pathway (such as *RELA*), apoptosis-related genes (*BCL2*, *BAX* and *CASP3*), and ce*ll* cycle (e.g., *MKI67*,* CCND1*,* CDK2*, and *CDKN1A*). The BEAS-2B cells cultured in 6-well plates were collected, and total RNA was isolated using the RNAeasy™ Animal RNA Isolation Kit with Spin Column (Beyotime Biotechnology, #R0026). Quantitative RNA (1 µg) underwent cDNA synthesis with SynScript^®^Ⅲ RT SuperMix (Tsingke, #TSK314S). The ArtiCanATM SYBR qPCR Mix was applied to conduct RT-qPCR analysis (Tsingke, #TSE501). All primers (Table S1) were synthesized by Thermo Fisher Scientific. We determined the relative expression levels of target genes using the 2^−ΔΔCt^ approach, with *glyceraldehyde-3-phosphate dehydrogenase (GAPDH)* employed as an internal control gene.

### Therapeutic effect of TCZ + DEX on asthma in mouse model

#### Experiments animals

Female BALB/c mice, aged 6–8 weeks, were obtained from Beijing Vital River Laboratory Animal Technology Co., Ltd. (Animal license number: SYXK (Lu) 2020 0009), were housed in an SPF environment for 7 days. The animals were kept on a 12 h light/dark cycle, with the temperature regulated at 23 ± 2℃ and relative humidity maintained at 40% ~ 60%. Sterile water and irradiated food were provided ad libitum. All animal care and experimental protocols were performed following the regulations set by the Animal Use and Care Committee of Qingdao University, and this experiment was approved by the Ethics Committee of Qingdao Medical College of Qingdao University (Ethical Number: QDU-AEC-2024770).

#### Asthma modeling and drug treatment

A total of 50 mice were randomly divided into five groups (*n* = 10 per group): control group (Control), model group (Model), TCZ-treated group (TCZ), combined treatment of TCZ and DEX (RunHong Pharm, Henan) group (TCZ + DEX), and DEX-treated group (DEX). Except for the control group, all mice were sensitized by intraperitoneal injection of 200 µL PBS containing 100 µg ovalbumin (OVA, Sigma, #A5503) and 1 mg Alhydrogel adjuvant (InvivoGen, #vac-alu) at day 0, 7, and 14. The control group mice were administered 200 µL of sterile normal saline *via* intraperitoneal injection. Starting from day 21, the control group mice were subjected to nebulized saline for 30 min, while the other mice were exposed to a 5% OVA solution for 30 min daily for 7 days. Simultaneously, from day 21, the TCZ group (5 mg/kg), TCZ (5 mg/kg) + DEX (1 mg/kg) group, and DEX group (2 mg/kg) mice received daily intraperitoneal injections of the corresponding treatment drugs for 7 days; saline was administered to both the control and model groups. The drug doses were determined according to previous studies [17–19] and our preliminary experiments. All mice were subjected to cervical dislocation and subsequent surgical procedures 24 h following the final stimulation. Animals were allocated to different analyses according to specific experimental requirements to ensure optimal sample integrity.

#### Evaluation of behavioral scores and pulmonary function

To evaluate behavioral performance, the behavioral scoring system was adapted from previously reported allergic airway inflammation models [20, 21] and modified to better reflect asthmatic manifestations, such as dyspnea and restlessness. The asthmatic behavior of the mice was observed within 10 min after the last provocation and scored as follows. The allergic response score was defined as follows: 0 points for no nose scratching or tickling, 1 point for 1–3 occurrences, 2 points for 4–6 occurrences, and 3 points for 7 or more occurrences. The respiratory symptom score was defined as: 0 points for no symptoms, 3 points for mild asthma symptoms, 6 points for shortness of breath or rapid breathing, rapid breathing rate, or restlessness, and 9 points for severe dyspnea. All behavioral assessments were independently performed by two blinded observers, and the mean score was used for statistical analysis.

The physiological properties of the mouse lungs were examined *via* lung function tests. Pentobarbital sodium was administered *via* intraperitoneal injection (50 mg/kg) to anesthetize the mice (Sigma, #P3761) and intubated, then placed in a supine position within a body tracing box. The other end of the tracheal intubation was connected to the lung function detection system (SCIREQ, FlexiVent FX), and the parameters were adjusted accordingly. The mice were fixed and operated according to the procedures following the manufacturer’s protocol, with subsequent data observation and recording. Once the data curve stabilized, data collection began. Lung function was measured using the flexiVent system in forced maneuver (FM) mode, and various lung function parameters were calculated using the flexiVent software.

#### Detection of inflammatory responses

Cytokine levels in BALF were measured as follows. The BALF was centrifuged at 1500 r/min for 10 min, and the precipitate was resuspended in PBS solution. The number of eosinophils and neutrophils was counted using an Auto Hematology Analyzer (Mindry, #BC-6800Plus). The collected supernatant was preserved for subsequent analyses. IL-4 (Fine Test, #EM0119) and IL-6 (Fine Test, #EN0121) were measured using ELISA kits, following the manufacturer’s protocols.

Serum and routine blood samples were collected and analyzed using the following procedure: mice were first anesthetized, followed by blood collection *via* orbital puncture. Blood samples were centrifuged at 2000 rpm for 10 min at 4 °C to isolate the serum. The serum was collected, and levels of IgE (Fine Test, #EM20006), IL-4 (Fine Test, #EM0119) and IL-6 (Fine Test, #EM021) were detected by ELISA following the manufacturer’s protocol. Additionally, blood samples were collected using microtainer vessels containing EDTA for blood routine analysis. The number of eosinophils and neutrophils was detected by the Auto Hematology Analyzer (Mindry, #BC-6800Plus).

The lung tissue was examined using hematoxylin-eosin (H&E) and Masson staining. The left lung tissue was cleaned with PBS, fixed in 4% paraformaldehyde, and preserved at 20–25 °C for 72 h. Subsequently, the tissue was embedded in paraffin, and paraffin sections were subjected to H&E and Masson staining to evaluate histopathological alterations. The degree of lung injury was semi-quantitatively scored using a score ranging from 0 (normal) to 4 (very severe), as previously described [22, 23]. The collagen fiber deposition rate in Masson-stained lung tissue was analyzed using ImageJ software.

#### Inflammatory pathway-related analysis

TNF-α, NF-κB, and phosphorylated NF-κB (p-NF-κB) immunohistochemical staining were used to analyze the inflammatory response and pathway expression in the asthma model. Lung tissue was fixed, paraffin-embedded, and sectioned into 4 µm slices. Subsequently, the sections underwent dewaxing and rehydration through a graded ethanol series. Antigen retrieval involved soaking the sections in boiling sodium citrate antigen recovery solution, followed by three washes with PBS. After incubation in a 3% H_2_O_2_ solution, blocking was performed using 5% bovine serum albumin (BSA). The NF-κB rabbit-derived monoclonal antibody (Cell Signaling Technology, #8242), p-NF-κB rabbit-derived monoclonal antibody (Cell Signaling Technology, #3033), and TNF-α mouse monoclonal antibody (Proteintech, #60291-1-lg), were used as primary antibodies and incubated with the sections at 4 °C for 12 h. Secondary antibody (ZSGB-BIO, #PV-6000) and 3,3’-Diaminobenzidine (DAB) staining were then performed. The primary antibody–positive signals were visualized under a light microscope. Relative quantification of the immunohistochemical staining images was performed by measuring the average optical density using ImageJ software.

The expressions of TNF-α, NF-κB, and p-NF-κB in lung tissues were detected by Western blot analysis. Lung tissue was lysed by radio-immunoprecipitation assay buffer (RIPA) and protein was extracted using a protein extraction reagent. Following separation by sodium dodecyl sulfate–polyacrylamide gel electrophoresis (SDS–PAGE), the proteins were transferred onto a PVDF membrane under optimized electrophoretic conditions. After transfer, the membrane was sealed at room temperature with rapid sealing solution for 30 min. After closure, the membrane was washed with tris-buffered saline containing Tween 20 (TBST). The primary antibodies, including TNF-α rabbit monoclonal antibody (Cell Signaling Technology, #11948T), NF-κB rabbit-derived monoclonal antibody (Cell Signaling Technology, #8242), p-NF-κB rabbit-derived monoclonal antibody (Cell Signaling Technology, #3033), GAPDH antibody (Bioss, #bsm-0978 M) and β-tubulin antibody (Proteintech, #80713-1-RR), were diluted at a ratio of 1:1000 and the membrane was incubated overnight at 4 °C. On the second day, the PVDF membrane was washed with TBST, and the appropriate secondary antibody, goat anti-rabbit IgG H&L antibody (Bioss, #bs-80295G-HRP), was diluted at a ratio of 1:10,000–1:50,000 and incubated with the membrane on a room temperature shaker for 1 h. The PVDF membrane was then washed thrice with TBST before Western blot analysis. The band intensity was analyzed using ImageJ software.

RT-qPCR served to assess the impact of drugs on gene expression in lung tissue. To assess the inflammatory response, transcript levels of *Il4*,* Il5*,* Il13* and *Tnf* were measured. Additionally, genes involved in the NF-κB signaling pathway (such as *Rela*) were also detected. All primers (Table S2) were synthesized by Thermo Fisher Scientific. Gene expression levels were quantified *via* the 2^−ΔΔCt^ approach, using *Actb* as the internal reference gene.

### Statistical analysis

Statistical analysis was performed using GraphPad Prism 10 software, and data were expressed as mean ± SD to reflect the variability among biological replicates. Differences among multiple groups were analyzed using one-way analysis of variance (ANOVA), followed by Tukey’s post hoc test for pairwise comparisons when statistically significant differences were observed. A p-value less than 0.05 was considered statistically significant (*p < 0.05, **p < 0.01, and ***p < 0.001).

## Results

### TCZ + DEX mitigates inflammatory toxicity in lung epithelial cells

**Fig. 2:**
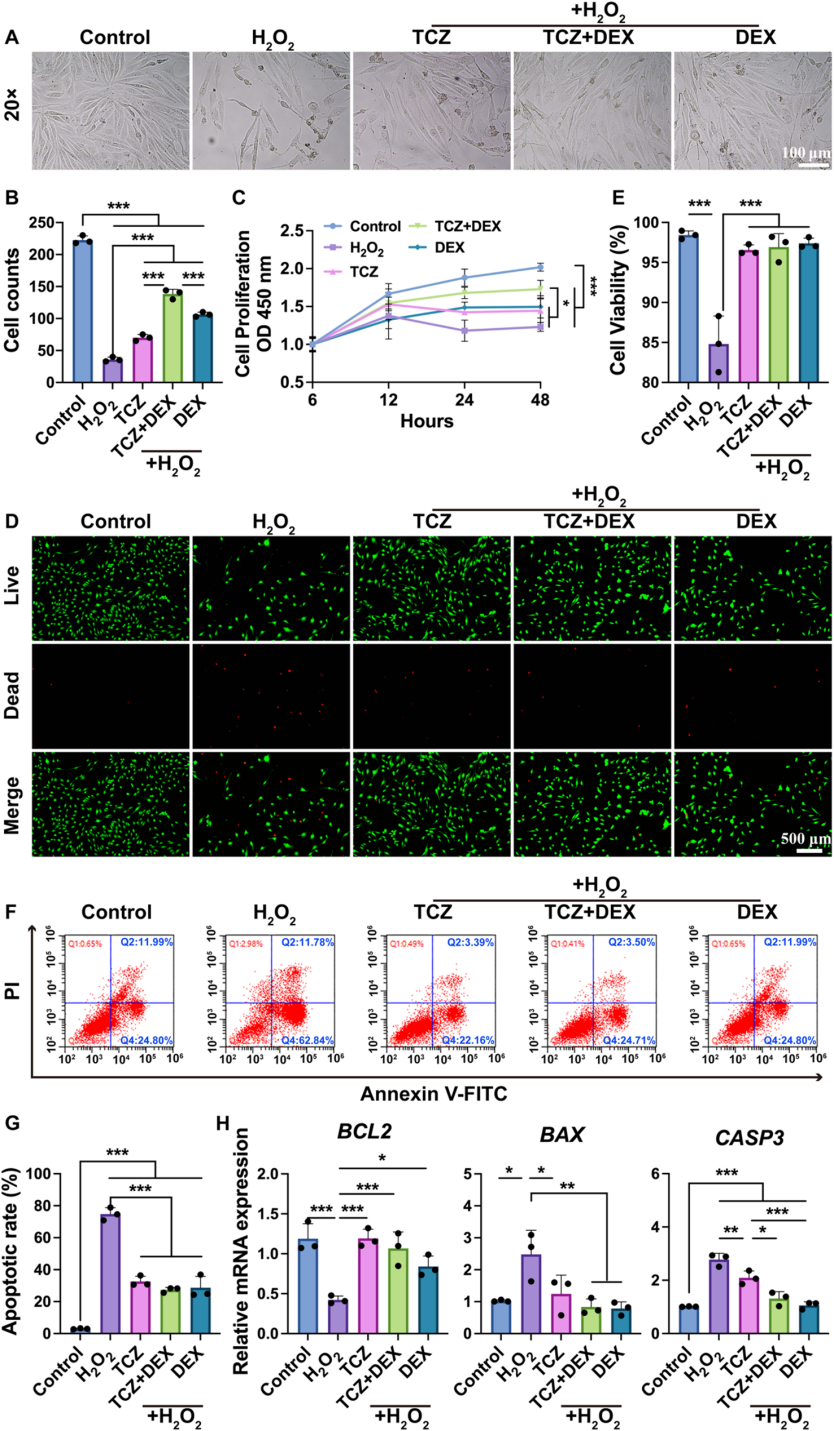
Proliferation, viability and apoptosis of BEAS-2B cells under the H_2_O_2_ treatment: (**A)** cell morphology, (**B**) quantification of cell counts from image (**A**), (**C**) cell proliferation, (**D**, **E**) Live/Dead cell staining and quantified cell viability, (**F**) cell apoptosis, (**G**) statistical analysis of apoptosis rate, and (**H**) *BCL2*,* BAX*,* CASP3* gene expression. *n*≥3, *p < 0.05, **p < 0.01, and ***p < 0.001

Given that asthma is a chronic inflammatory respiratory condition [24], this study examined the protective role of TCZ + DEX in cells exposed to an oxidative stress microenvironment under in vitro H_2_O_2_-stimulated inflammation. As shown in Fig. 2A**/**2B, H_2_O_2_ treatment significantly reduced cell numbers relative to the control group. This suggests that the mimicked inflammatory microenvironment inhibited cell proliferation. After drug treatment, cell counts increased significantly in all groups (TCZ, TCZ + DEX, and DEX), with the TCZ + DEX group exhibiting a notably higher cell count than the TCZ and DEX groups (increases of approximately 97.6% and 30.1%, respectively). This suggests that TCZ + DEX exerted a stronger protective effect on cell growth compared with monotherapy. CCK-8 results further corroborated these trends (Fig. 2C). In addition, while cell viability in the control group continuously increased over 48 h, cell proliferative activity in the H_2_O_2_ group was significantly reduced, indicating severe impairment of proliferation. Although TCZ or DEX treatment alone improved cell proliferation, the TCZ + DEX group demonstrated a more pronounced protective effect in restoring cell proliferation.

H_2_O_2_, as a representative agent of oxidative stress, induces cellular damage by activating intracellular redox reactions and ultimately leads to apoptosis [25]. To evaluate the impact of TCZ + DEX on cellular apoptosis, live/dead cell staining and flow cytometry assays were performed. Live/dead staining results revealed that the control group exhibited nearly 100% cell survival. Although the H_2_O_2_ group showed a significant decrease in survival rate (Fig. 2D, E), drug treatments (e.g., TCZ, TCZ + DEX, and DEX) improved cell survival to levels comparable to the control group. Flow cytometry analysis supported the above finding (Fig. 2F, G). The control group displayed minimal apoptosis, whereas a substantial elevation in apoptosis (approximately 74.8%) was observed in the H_2_O_2_ group. Treatment with TCZ or DEX reduced the apoptosis rate to approximately 32.6% and 28.6%, respectively. The TCZ + DEX group demonstrated an apoptosis rate of around 27.34%, achieving an outcome comparable to that of DEX, despite the reduced DEX dosage. Gene expression analysis (Fig. 2H) at the molecular level revealed a notable downregulation of the anti-apoptotic gene *BCL2* and upregulation of the pro-apoptotic markers *BAX* and *CASP3* in the H_2_O_2_-treated group relative to the control. The TCZ + DEX and DEX groups showed *CASP3* expression reductions of approximately 52.9% and 55.0%, respectively, compared to the H_2_O_2_ group—more substantial than the 24.8% decrease observed in the TCZ group. Moreover, the expression levels of all genes involved in apoptosis in the TCZ + DEX combination group were comparable to those observed in the DEX monotherapy group. These findings suggest that TCZ + DEX mitigated inflammation-induced cell damage and produced protective effects comparable to DEX while using a lower DEX dose. This may result from the complementary actions of TCZ’s anti-inflammatory properties, which confer protective effects in models of acute lung injury and ulcerative colitis by inhibiting inflammation and cell apoptosis [26, 27], and DEX’s classic anti-inflammatory and antioxidant effects.

### TCZ + DEX ameliorates oxidative stress and mitochondrial function of cells

**Fig. 3:**
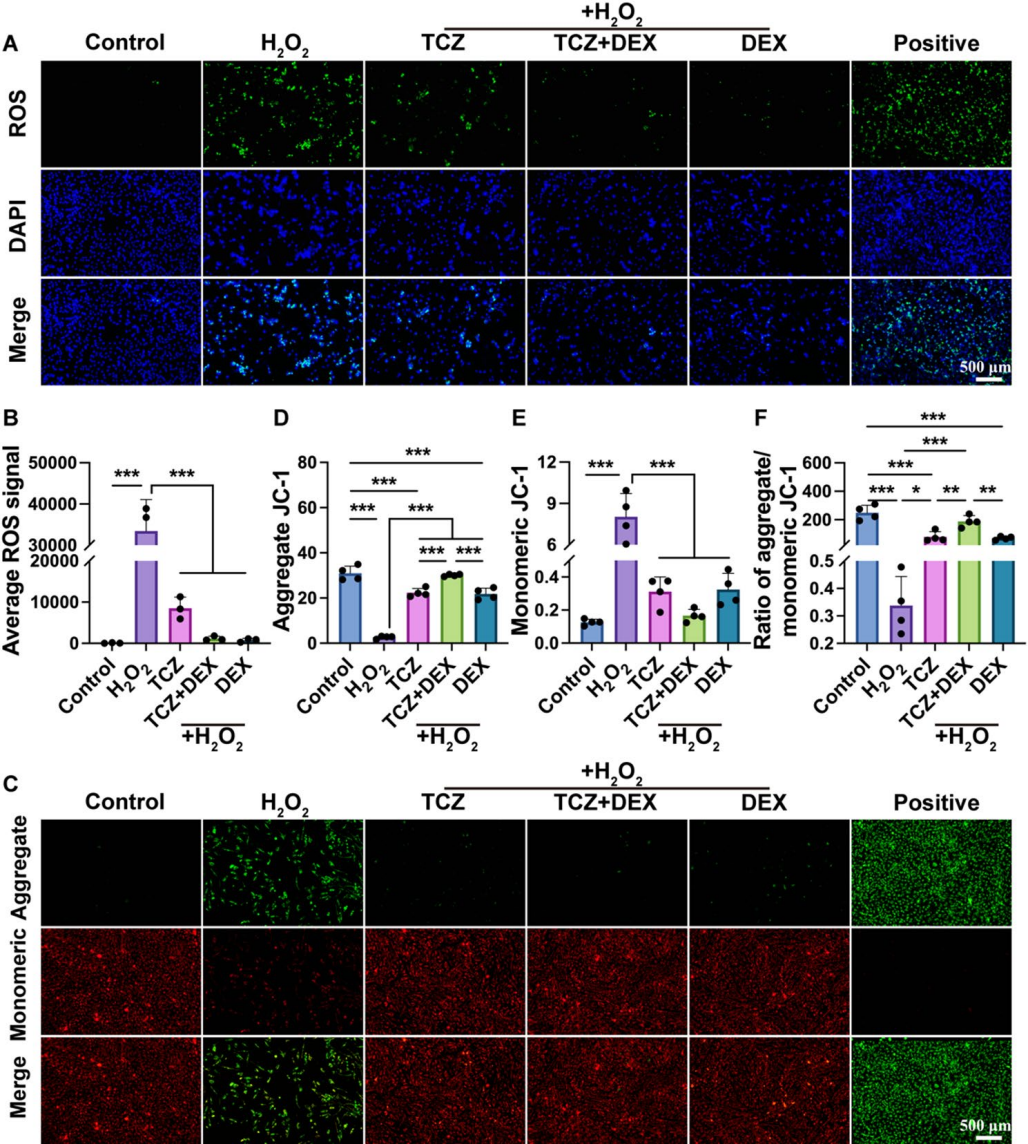
H_2_O_2_-induced changes of ROS levels and JC-1 in BEAS-2B cells: (**A) **intracellular ROS expression, (**B**) quantified ROS expression from image (**A**), (**C**) mitochondrial membrane potential, (**D**-**F**) quantification of JC-1 aggregate, JC-1 monomer, and JC-1 ratio. *n*≥3, *p < 0.05, **p < 0.01, and ***p < 0.001

Oxidative stress affects ΔΨm by increasing intracellular ROS production, thereby regulating apoptosis [28, 29]. Excessive ROS accumulation can impair mitochondrial function, disrupt ΔΨm stability, and induce cellular metabolic disturbances. Normal ΔΨm is crucial for cellular function; its loss hinders mitochondrial energy synthesis, triggering apoptotic signaling pathways, including the mitochondrial-dependent pathway, ultimately leading to cell apoptosis [30]. To elucidate how oxidative stress influences ΔΨm *via* ROS accumulation and induces apoptosis, we evaluated ROS generation and mitochondrial ΔΨm. ROS analysis (Fig. 3A, B) revealed that the H_2_O_2_ group exhibited significantly higher average ROS fluorescence intensity than the control group, while the TCZ, TCZ + DEX, and DEX groups showed ROS intensity reductions of 74.5%, 97.7%, and 96.4%, respectively, compared to the H_2_O_2_ group. While all three treatments demonstrated similar efficacy, the TCZ + DEX combination achieved reductions comparable to DEX despite using a lower DEX dose, highlighting its more pronounced suppression of ROS accumulation. Although TCZ and DEX individually mitigate oxidative stress, their combined administration may exert complementary effects that reduce drug burden and minimize side effects, offering a promising clinical therapeutic strategy.

ΔΨm was assessed *via* JC-1 staining (Fig. 3C), where JC-1 aggregates indicate high ΔΨm (Fig. 3D) and JC-1 monomers reflect ΔΨm reduction (Fig. 3E). The H_2_O_2_ group exhibited significantly lower JC-1 aggregate levels and higher monomer levels compared to the control group. This indicates mitochondrial depolarization and ΔΨm loss. In contrast, treatment with TCZ, DEX, and TCZ + DEX restored JC-1 aggregation and reduced monomer levels, suggesting partial mitigation of mitochondrial damage. Among these treatments, TCZ + DEX demonstrated superior ΔΨm restoration, with JC-1 aggregate levels approximately 34.6% and 37.7% higher than in the TCZ and DEX groups, respectively. Furthermore, JC-1 monomer levels in the TCZ + DEX group were approximately 46.6% and 48.7% lower than in the TCZ and DEX groups. The JC-1 ratio, a key indicator of ΔΨm stability (Fig. 3F), was significantly reduced in the H_2_O_2_ group but showed an increase in the TCZ, DEX, and TCZ + DEX groups. Notably, the TCZ + DEX group exhibited JC-1 ratios approximately 137.3% and 168.3% higher than in the TCZ and DEX groups, respectively, suggesting enhanced mitochondrial function restoration through complementary or additive effects of the combined treatment. These findings collectively underscore the superior efficacy of TCZ + DEX combination therapy in safeguarding cellular function through enhanced ΔΨm recovery.

Previous studies have demonstrated that TCZ treatment effectively reduces oxidative stress biomarkers in the serum of rheumatoid arthritis patients [31]. We further investigated the protective effects of TCZ + DEX combination therapy against oxidative stress–induced cellular damage and mitochondrial dysfunction. Specifically, TCZ + DEX reduced ROS accumulation, restored ΔΨm, enhanced mitochondrial protection, improved cellular physiological status, and mitigated oxidative stress–induced damage and apoptosis. These findings emphasize the promise of combination therapy for the treatment of oxidative stress–related diseases, particularly its more pronounced protective effects compared with monotherapy. Overall, this study provides a novel perspective on TCZ + DEX combination therapy for oxidative stress–related diseases. Future studies employing transcriptomic or metabolomic approaches to clarify the molecular processes underlying the observed combined regulation of mitochondrial homeostasis may help optimize therapeutic strategies for oxidative injury.

### TCZ + DEX attenuates NF-κB activation and inflammatory responses in H_2_O_2_-induced BEAS-2B cells

**Fig. 4:**
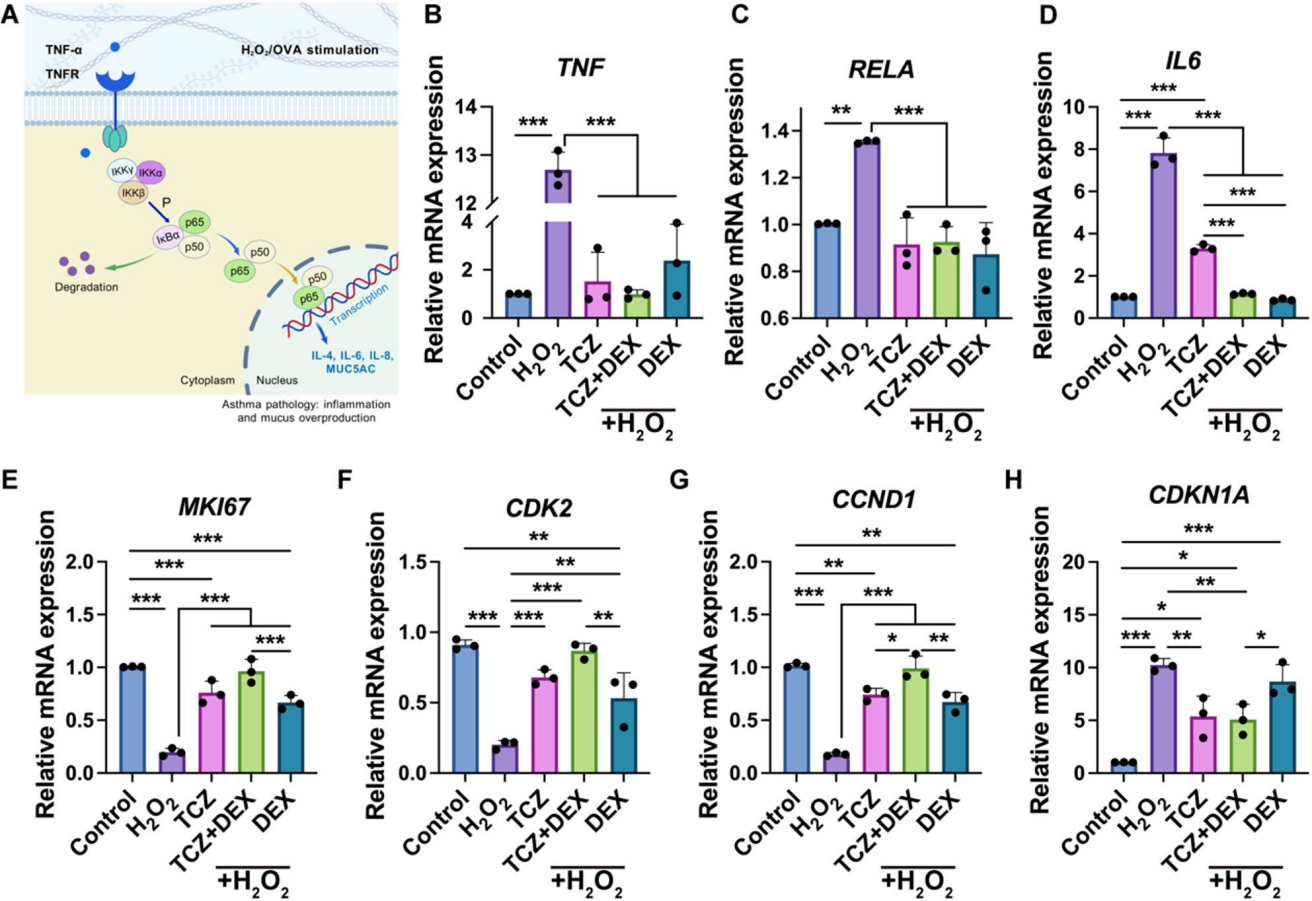
H_2_O_2_ induced the expression of inflammatory factors, NF-κB–related genes, and cell cycle-related genes in BEAS-2B cells: (**A)** schematic diagram of the NF-κB signaling pathway mechanism, (**B**) *TNF* gene expression, (**C**) *RELA* gene expression, (**D**) *IL6* gene expression, (**E**) *MKI67* gene expression, (**F**) *CDK2* gene expression, (**G**) *CCND1* gene expression, and (**H**) *CDKN1A* gene expression. *n* = 3, *p < 0.05, **p < 0.01, and ***p < 0.001

The reduction in inflammatory mediators may be associated with suppressed NF-κB activation (Fig. 4A). Allergens, inflammatory factors, or oxidative stress stimuli activate the TNF-α receptor (TNFR), which subsequently triggers the IKK complex (e.g., IKKα, IKKβ, and IKKγ), resulting in the degradation of IκBα and nuclear translocation of NF-κB subunits (p65/p50). This translocation promotes the transcription of inflammatory mediators, thereby exacerbating the inflammatory response [32, 33]. As a core transcriptional regulator during oxidative stress and inflammation-induced cellular processes, NF-κB serves as a key regulator of gene expression associated with cell survival, immune responses, and inflammation processes [34]. To investigate the effects of TCZ + DEX on inflammatory cytokines and signaling pathways, qPCR was used to assess changes in *IL6*,* TNF*, and *RELA* expression. The qPCR results (Fig. 4B-D) revealed that H_2_O_2_ treatment markedly upregulated the mRNA expression levels of *TNF*,* RELA*, and *IL6*, indicating a robust inflammatory response. All treatment groups attenuated these changes. Specifically, the TCZ, DEX, and TCZ + DEX groups significantly inhibited *TNF* expression, with reductions of approximately 88%, 81.2%, and 92.2%, respectively, compared with cells treated with H_2_O_2_. There were no statistically significant variations among the different treatment conditions, indicating that all three treatments effectively suppressed *TNF* expression. Regarding *RELA*, the TCZ, DEX, and TCZ + DEX groups reduced *RELA* levels by approximately 32.3%, 35.5%, and 31.6%, respectively, relative to the H_2_O_2_ group, with no significant variation detected between groups. For *IL6*, the TCZ + DEX and DEX groups reduced its expression by approximately 88.9% and 85.2%, respectively, compared to the H_2_O_2_ group. Both reductions were significantly greater than the around 57.7% reduction observed in the TCZ group. This pattern aligns with the previously observed modulation of the NF-κB pathway–related gene expression (Fig. 4A). These findings highlight the potential advantages of TCZ + DEX combination therapy, particularly its enhanced ability to reduce inflammatory cytokine expression. This study suggests that TCZ and DEX may influence NF-κB–related signaling through distinct but complementary molecular processes. This provides a basis for further investigation into the mechanisms underlying their combined therapeutic effects.

Previous studies suggest that NF-κB is critically involved in modulating immune activity and inflammatory signaling. It triggers the production of cytokines such as TNF-α and IL-6, consequently enhancing inflammatory responses [35, 36]. The current investigation sought to refine inflammatory regulation through a combinatorial therapeutic approach. The primary objective was to evaluate the impact of TCZ + DEX on the inflammatory cascade while concurrently diminishing the DEX dosage, aiming to preserve or enhance therapeutic efficacy. qPCR analysis revealed that both TCZ and DEX, individually and in combination, significantly attenuated H_2_O_2_-induced inflammatory responses. The TCZ + DEX combination exhibited potent anti-inflammatory properties, supporting the potential of this regimen to maintain therapeutic efficacy outcomes while helping to reduce overall glucocorticoid (GC) exposure, which may be relevant for future dosing strategies.

### TCZ + DEX combination therapy mitigates the inhibitory effects of DEX on the cell cycle

In addition to its anti-inflammatory effects, a critical aspect of assessing a drug’s safety profile lies in its influence centers on proliferative kinetics and cell cycle progression. Key regulatory proteins in these processes encompass *MKI67*,* CDK2*,* CCND1*, and *CDKN1A* [37, 38]. Notably, *MKI67* is a marker for cellular proliferative activity, whereas *CDK2* and *CCND1* play pivotal roles in orchestrating the G1/S phase transition. In contrast, *CDKN1A* functions as a cell cycle inhibitor. To further evaluate whether the combination of TCZ and DEX impacts cell proliferation, we examined the expression levels of relevant genes. While DEX possessed significant anti-inflammatory properties, prolonged or high-dose use impeded cell proliferation and potentially disrupted cellular function by inducing cell cycle arrest [39, 40]. This conclusion aligns with our research findings, prompting an exploration of the combined effects of combining TCZ with lower doses of DEX in both inflammation control and cell cycle regulation. Results indicated that, the H_2_O_2_-treated group exhibited a significant decrease in the expression of *MKI67*,* CDK2*, and *CCND1*, alongside a marked increase in *CDKN1A* expression, compared with the control group. These alterations suggest that H_2_O_2_-induced oxidative stress inhibited cell proliferation and induced cell cycle arrest (Fig. 4E-H). Treatment with TCZ led to the recovery of *MKI67*,* CDK2*, and *CCND1* mRNA expression levels to approximately 75.6%, 74.6%, and 72.8% of those observed in the control group, respectively, and substantially reduced *CDKN1A* expression (approximately 47.5% lower than the H_2_O_2_ group). The TCZ + DEX combination treatment exhibited even more robust restorative effects, restoring *MKI67*,* CDK2*, and *CCND1* expression to levels approaching those of the control group, and significantly lowering *CDKN1A* expression. In contrast, DEX alone only restored *MKI67*,* CDK2*, and *CCND1* expression to approximately 75.6%, 74.6%, and 72.8% of the control group, with *CDKN1A* expression levels exhibiting no significant difference from the H_2_O_2_ group. This suggests that the TCZ + DEX combination treatment exhibited more potent restorative effects in coordinately regulating the cell cycle and alleviating the cell proliferation inhibition potentially induced by DEX.

### TCZ + DEX promotes lung function recovery in asthmatic mice

**Fig. 5:**
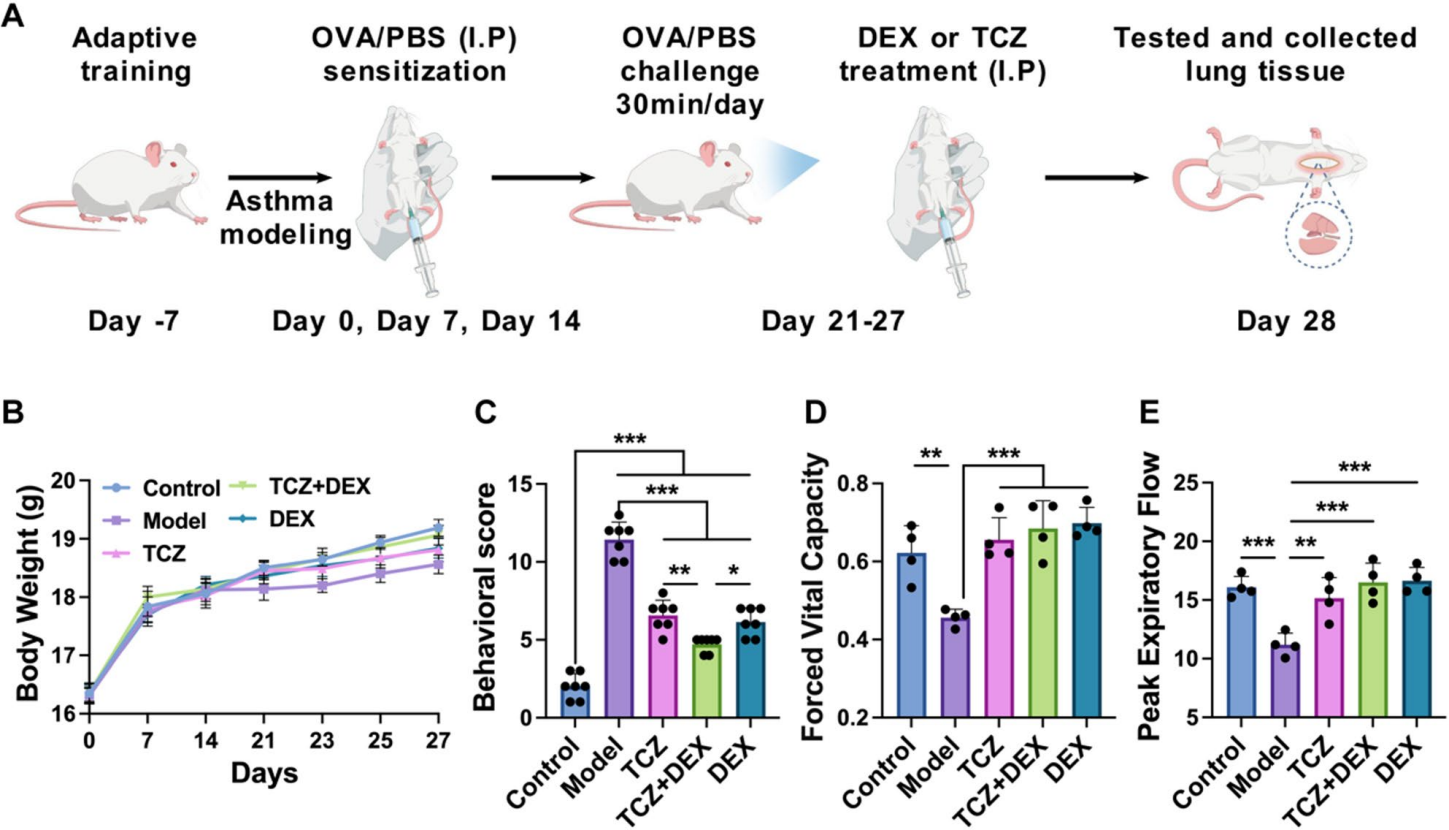
OVA-induced asthma mice model establishment and lung function evaluation: (**A)** asthma mice model establishment, (**B**) body weight change, (**C**) asthma behavioral scoring, (**D**) Forced Vital Capacity, and (**E**) Peak Expiratory Flow. *n* = 4 to 8, *p < 0.05, **p < 0.01, and ***p < 0.001

In the animal experiments, we further validated the findings from the cellular studies. Throughout the experimental duration, all mouse cohorts displayed normal mental status and demonstrated a degree of weight gain. Upon administration of drugs and nebulization treatment, body weight fluctuations were observed across all groups, subsequently followed by a trend toward stabilization. The overall weight change patterns were consistent among groups, with no statistically significant differences observed (Fig. 5B), indicating that OVA, TCZ, and DEX did not exert a notable impact on body weight in mice. OVA sensitization and challenge are known to induce characteristic asthma symptoms in mice, including wheezing, restlessness, and abdominal breathing, accompanied by a decline in pulmonary function [41]. An asthma model in mice was developed by OVA sensitization and challenge, employing a method consistent with previous protocols. Disease severity and therapeutic efficacy were assessed through behavioral scoring and pulmonary function evaluation. Asthmatic model mice exhibited a markedly elevated behavioral score relative to the control group, characterized by obvious symptoms including wheezing, nasal scratching, ear rubbing, and abdominal respiration (Fig. 5C). In contrast, the TCZ, DEX, and TCZ + DEX treatment groups exhibited markedly lower behavioral scores compared to the model group, suggesting that each treatment regimen effectively alleviated asthma-related symptoms in mice. Notably, the TCZ + DEX group exhibited superior therapeutic effects compared to the single-drug groups.

Lung function is critical for assessing asthma, as it reflects airway constriction and pulmonary dysfunction [42, 43]. The results indicated that the OVA-induced asthma mouse model displayed notable pulmonary dysfunction. FVC (Fig. 5D) and PEF (Fig. 5E) declined by about 26.7% and 30.5%, respectively, when compared with controls, supporting the validity of the model. Pulmonary function tests were performed to further evaluate the effects of different treatments. Both TCZ and DEX treatments led to significant improvements in lung function. TCZ treatment resulted in an approximately 44.4% increase in FVC and an approximately 36.4% increase in PEF compared to the model group. Similarly, DEX treatment caused an approximately 53% increase in FVC and an approximately 48.8% increase in PEF. The TCZ + DEX combination therapy exhibited comparable effects to the monotherapies, improving FVC by approximately 50% and PEF by 47.5% compared to the model group. Through behavioral scoring and pulmonary function assessments, we found that TCZ + DEX treatment significantly improved the clinical symptoms and pulmonary function of asthmatic mice, particularly when the DEX dosage was halved, confirming its effect on alleviating clinical symptoms and improving pulmonary function in asthmatic mice. Although airway resistance and compliance measurements were not performed in this study due to equipment limitations, FVC and PEF provided reliable information on overall ventilatory function and airflow improvement, adequately reflecting the therapeutic effects of TCZ + DEX.

### TCZ + DEX reduces pulmonary inflammation in asthmatic mice

**Fig. 6:**
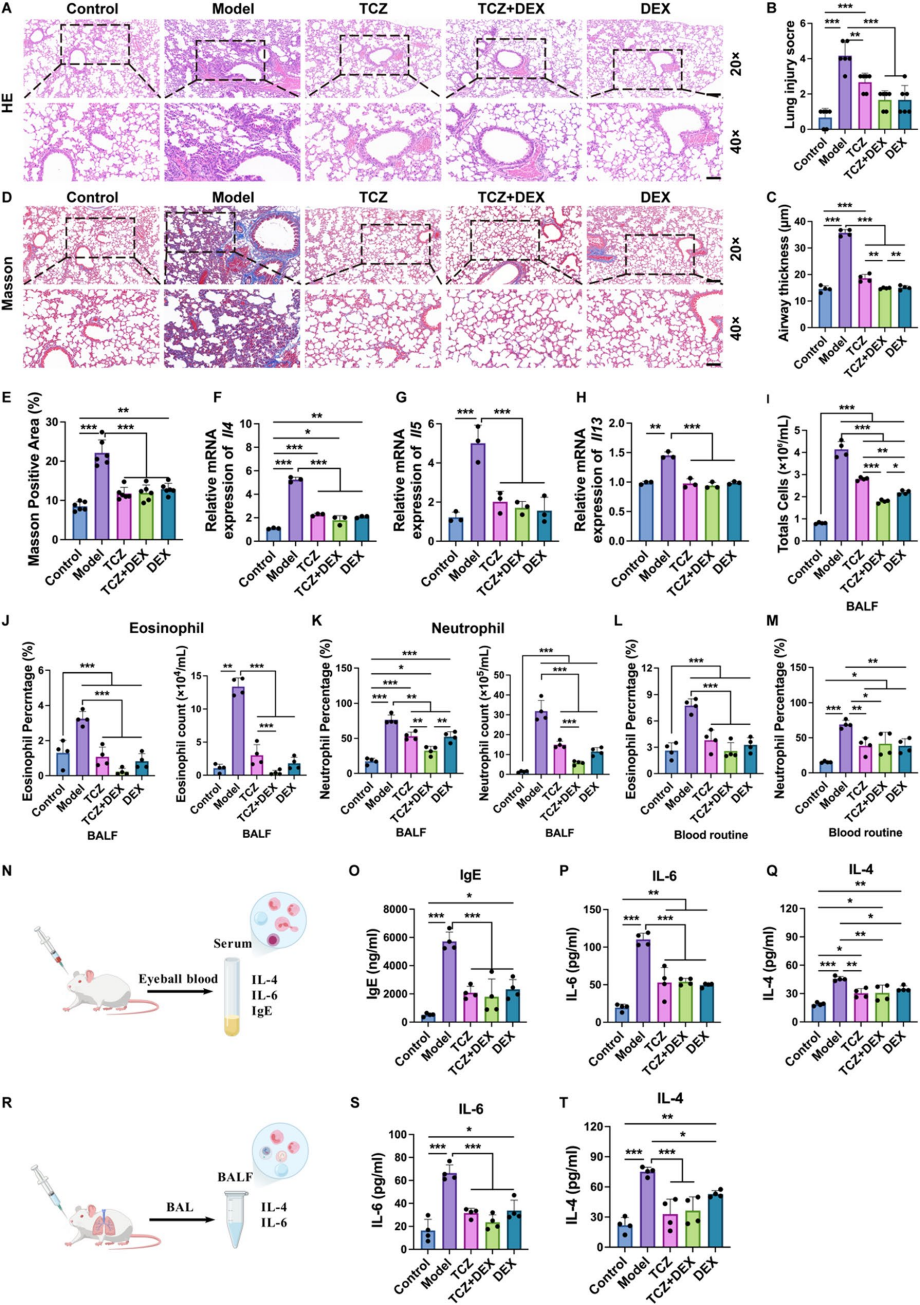
Histopathological observation and inflammatory factor expression in OVA-Induced asthma mice: (**A)** H&E staining of the lung sections, 20×, scale bar, 100 μm; 40×, scale bar, 50 μm, (**B**) lung injury score, (**C**) quantification of airway thickness, (**D**) Masson staining of the lung sections, 20×, scale bar, 100 μm; 40×, scale bar, 50 μm, (**E**) quantitative analysis of Masson staining, (**F**) pulmonary *Il4* gene expression, (**G**) pulmonary *Il5* gene expression, (**H**) pulmonary *Il13* gene expression, (**I**) total cell count in BALF, (**J**) analysis of eosinophils in BALF, (**K**) analysis of neutrophils in BALF, (**L**) percentage analysis of eosinophils in blood routine, (**M**) percentage analysis of neutrophils in blood routine, (**N**) serum ELISA diagram, (**O**-**Q**) expression of IgE, IL-6 and IL-4 in serum, (**R**) BALF ELISA diagram, and (**S**, **T**) expression of IL-4 and IL-6 in BALF. *n* = 4 to 6, *p < 0.05, **p < 0.01, and ***p < 0.001

Histological alterations in lung tissue were observed through H&E staining. Relative to the control group, model group mice exhibited significantly aggravated lung inflammation, characterized by pronounced infiltration of inflammatory cells surrounding the airways, disordered arrangement of lung tissue, thickening of bronchial walls, and inflammatory cell infiltration. Treatment with TCZ, DEX, and TCZ + DEX alleviated the pathological symptoms of the lungs to varying degrees (Fig. 6A). Pathological lung injury scores recorded in the model group exhibited a significant elevation relative to the control group. Following intervention, the average injury score was approximately 2.67 in the TCZ group (36.0% lower than the model group), while both the DEX and TCZ + DEX groups showed a greater reduction, with an average score of 1.67 (~ 60.0% decrease). There was no significant variation between the two treatment groups, suggesting that they have similar therapeutic effects (Fig. 6B). The airway thickness analysis results indicated that compared to the control group, the airway wall thickness was significantly increased in the model group (Fig. 6C). All treatment groups showed varying degrees of reduction in airway wall thickness compared to the model group. Specifically, the TCZ group, the TCZ + DEX combination treatment group, and the DEX group exhibited reductions of approximately 47.9%, 58.4%, and 57.9%, respectively, relative to the model group. The results demonstrate that the TCZ + DEX combination treatment showed a numerically greater trend in improving airway wall thickness. Masson staining was employed to observe collagen deposition in the airways of the lungs. Compared to the control group, the model group exhibited increased collagen deposition in the lung tissue, whereas the treatment groups showed reductions in collagen deposition (Fig. 6D). Semi-quantitative findings demonstrated a marked elevation in the percentage of collagen deposition in the model group versus the control group (Fig. 6E). After treatment, the average collagen deposition rate in the TCZ monotherapy group was approximately 46.9% lower than that in the model group, although it was still markedly elevated relative to the controls. The average collagen deposition rates in the DEX group and TCZ + DEX combination treatment group were approximately 41.4% and 46.0% lower, respectively, compared to the model group. While the DEX group showed a collagen deposition rate higher than the control group, the TCZ + DEX group restored collagen deposition to levels comparable to those of the control group. No significant differences were observed between the treatment groups.

qPCR results showed a marked elevation in *Il4*, *Il5* and *Il13* transcript levels in OVA-induced asthmatic mice, reflecting the activation of Th2-driven immune mechanisms typically observed in asthma. *Il4*, *Il5* and *Il13* are typical Th2 cytokines that play pivotal roles in airway inflammation, airway hyperresponsiveness, and the recruitment of eosinophils [4]. Compared to the control group, the model group exhibited significantly elevated *Il4*, *Il5* and *Il13* expression (Fig. 6F-H). Subsequent TCZ treatment reduced the expression of *Il4*, *Il5* and *Il13* by approximately 57.0%, 59.8% and 32.7%, respectively, relative to the model group. Both DEX and TCZ + DEX treatments significantly reduced in *Il4*,* Il5* and *Il13* expression compared with the model group, with no significant differences observed between these two groups. This suggests that both treatments similarly reduced the levels of these inflammatory factors. The TCZ monotherapy group showed weaker results compared to the other treatment groups, suggesting that its monotherapy effect is limited.

In the asthmatic mouse model, airway inflammation is often accompanied by the infiltration of eosinophils and neutrophils [44, 45]. The total cell count in the BALF was analyzed to evaluate the overall inflammatory cell infiltration in the lungs (Fig. 6I). Compared with the control group, the total cell count in the model group was significantly increased. All treatment groups reversed this trend, with the TCZ monotherapy group, TCZ + DEX combination therapy group, and DEX group showing reductions in total cell count by approximately 32.3%, 56.7%, and 47.0%, respectively, compared to the model group. Moreover, the TCZ + DEX combination therapy demonstrated the most significant effect in suppressing inflammatory cell recruitment. In the model group, the proportion of eosinophils (Fig. 6J) and neutrophils (Fig. 6K) in the BALF was significantly elevated, reflecting the inflammatory response in the airways. The number of eosinophils and neutrophils in the TCZ and DEX groups was both significantly reduced compared to the model group. Notably, the eosinophil proportion in the TCZ + DEX group exhibited a marked reduction of approximately 92.9% relative to the model group. Furthermore, the efficacy of the combination regimen in reducing eosinophils exceeded that of TCZ monotherapy. The TCZ + DEX group exhibited the most pronounced reduction in neutrophil proportion, decreasing by approximately 57.7% compared to the model group. This result demonstrates a clear anti-inflammatory effect that was significantly superior to the monotherapy groups. Hematological analysis further revealed a significant increase in the proportion of eosinophils (Fig. 6L) and neutrophils (Fig. 6M) in the blood of model group mice, indicating the occurrence of a systemic inflammatory response. The quantity of eosinophils and neutrophils in the TCZ and DEX groups showed a notable reduction relative to the model group. The TCZ + DEX combination group exhibited a significant decrease in eosinophil and neutrophil counts by approximately 66.7% and 40%, respectively, relative to the model group, with no significant differences detected among the treatment groups. In this study, the OVA-induced asthma model showed significant infiltration of both eosinophils and neutrophils. The increase in neutrophils may be related to the acute inflammatory response triggered by the high-dose OVA, a phenomenon that has been reported in some studies [46, 47].

ELISA analysis of serum (Fig. 6N) and BALF (Fig. 6R) further corroborated these findings. Previous studies have shown that IgE is a key marker of allergic reactions and is closely associated with airway inflammation, eosinophil infiltration, and airway hyperresponsiveness [48]. In this study, a notable elevation in plasma IgE levels was fund in the asthma group, suggesting a clear allergic inflammatory response (Fig. 6O). Treatment with TCZ, TCZ + DEX, and DEX alone reduced IgE levels by approximately 63.5%, 68.5%, and 59.3%, respectively, relative to the model group. These findings indicate that all treatment regimens effectively inhibit the elevation of IgE, thereby alleviating airway allergic reactions. Plasma levels of IL-6 (Fig. 6P) and IL-4 (Fig. 6Q) were markedly increased in the model group. In the TCZ, TCZ + DEX, and DEX alone treatment groups, IL-6 levels were reduced by approximately 52.0%, 51.0%, and 55.2%, respectively, compared to the model group. IL-4 levels were reduced by approximately 33.4%, 32.7%, and 23.3%, respectively. Statistical analysis revealed no significant differences between the treatment groups. BALF analysis demonstrated a notable rise in IL-4 (Fig. 6S) and IL-6 (Fig. 6T) concentrations in the model group. Treatments with TCZ, DEX, and their combination significantly reduced these cytokines, with IL-4 reductions of 56.2%, 29.6%, and 51.5%, and IL-6 reductions of 59.7%, 68.9%, and 65.9%, respectively. Differences among treatment groups were not statistically significant.

This study revealed that TCZ combined with DEX effectively alleviated inflammation in mice with asthma, as indicated by diminished inflammatory cell infiltration and decreased concentrations of IL-4, IL-5, and IL-6. This result aligns with earlier reports and reinforces the therapeutic promise of TCZ not only in asthma management but also in treating other inflammatory lung disorders [17, 49, 50]. Additionally, the combination therapy also reduced eosinophil and neutrophil infiltration, offering a more efficient and safer option for managing asthma and other inflammatory conditions compared to monotherapy.

### TCZ + DEX is associated with reduced NF-κB activation and attenuated airway inflammation in asthmatic mice

**Fig. 7:**
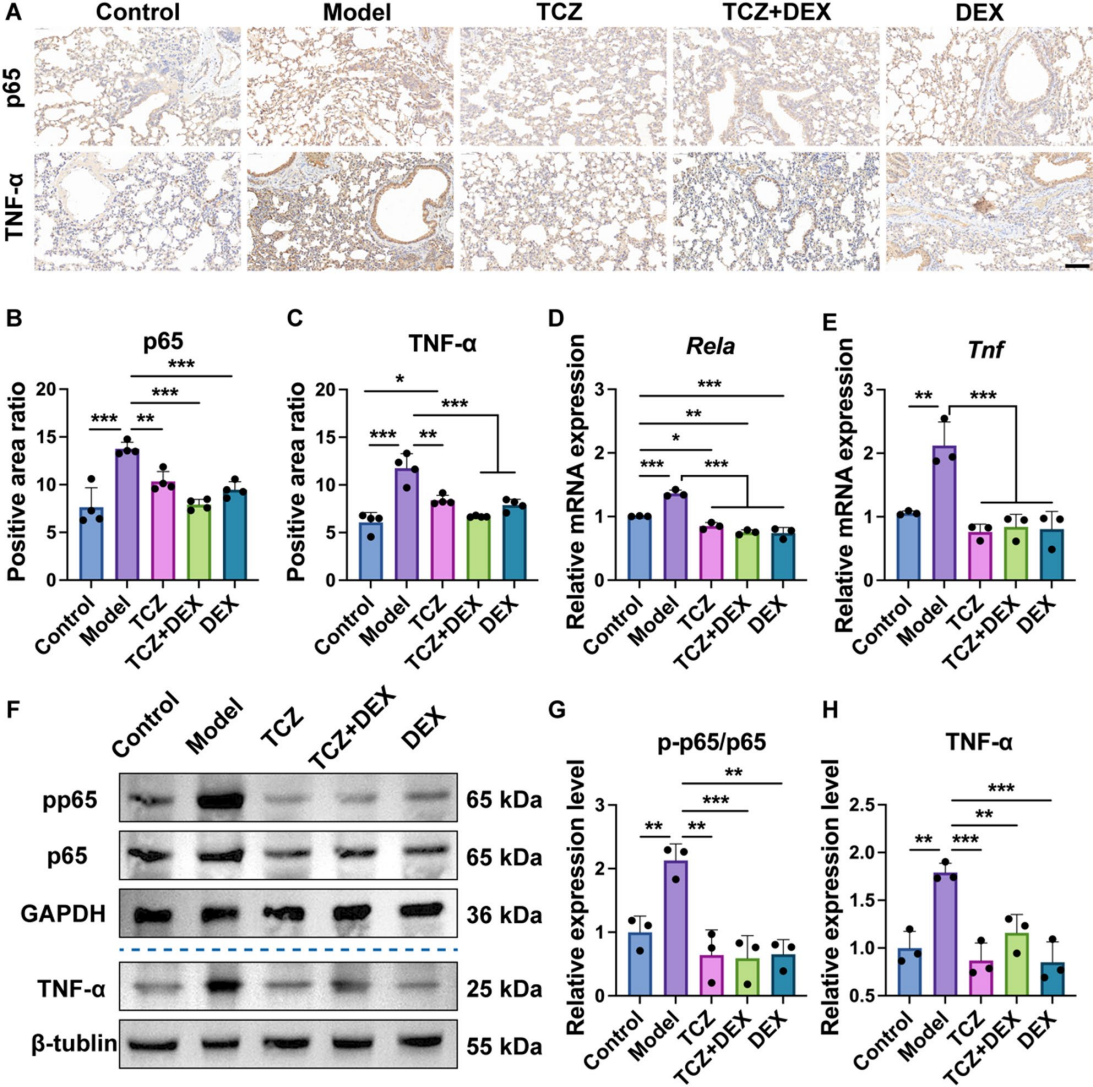
TNF-α and NF-κB expression in the pulmonary tissues of mice with asthma induced by OVA: (**A)** p65 and TNF-α immunohistochemistry, 40×, scale bar, 100 μm, (**B**) positive area ratio of p65, (**C**) positive area ratio of TNF-α, (**D**) pulmonary *Rela* gene expression, (**E**) pulmonary *Tnf* gene expression, (**F**) protein expression of pp65, p65, and TNF-α in pulmonary tissue detected by Western blotting, (**G**) relative expression level of pp65/p65, and (**H**) relative expression level of TNF-α. *n* = 3 to 4, *p < 0.05, **p < 0.01, and ***p < 0.001

Regarding molecular mechanism, our findings suggest that the anti-inflammatory effects of TCZ + DEX treatment are associated with reduced NF-κB activation. Excessive activation within the NF-κB pathway plays a pivotal role in airway inflammatory responses and tissue damage in asthma and related chronic inflammatory disorders [51, 52]. Immunohistochemical analysis (Fig. 7A) revealed markedly elevated levels of p65 and TNF-α expression in pulmonary tissues from OVA-induced asthmatic mice. In this study, the model group exhibited a significant upregulation of p65 (Fig. 7B) and TNF-α (Fig. 7C) expression compared to the control group, with increases of approximately 80.0% and 93.7%, respectively. After treatment, the TCZ group exhibited reductions of approximately 24.8% in p65 and 28.6% in TNF-α positive areas relative to the model group. In the DEX group, p65 and TNF-α levels were reduced by approximately 31.5% and 43.0%, respectively. The TCZ + DEX group also demonstrated significant inhibitory effects on p65 and TNF-α expression, with reductions of approximately 42.6% and 32.9%, respectively, relative to the model group. In comparison with TCZ monotherapy, the inhibitory effect of TCZ + DEX on TNF-α expression was comparable to that of DEX alone, both of which restored expression levels to those similar to the control group. These results suggest that combination therapy effectively alleviated airway inflammation while maintaining therapeutic efficacy at a reduced DEX dosage.

Further confirmation was obtained *via* qPCR, which showed that the mRNA levels of *Rela* (Fig. 7D) and *Tnf* (Fig. 7E) were significantly elevated in the model group. After treatment, *Rela* expression in the TCZ group decreased by approximately 37.7% relative to the model group. The DEX and TCZ + DEX groups exhibited decreases of approximately 45.5% and 44.8%, respectively, in *Rela* expression relative to the model group. *Tnf* expression was reduced by approximately 64.2%, 60.3%, and 62.0% in the TCZ, TCZ + DEX, and DEX groups, respectively, compared to the model group. Western blot results (Fig. 7F) showed a notable elevation in both the pp65/p65 ratio and TNF-α protein levels within pulmonary tissue of asthmatic mice. Treatment with TCZ, DEX, and their combination led to reductions in pp65/p65 levels by approximately 69.8%, 69.4%, and 72.3%, respectively, relative to the model group (Fig. 7G). TNF-α protein expression was reduced by approximately 51.4%, 52.5%, and 35.2% in the TCZ, DEX, and TCZ + DEX groups, respectively, when compared to the model group (Fig. 7H). No statistically significant variations in protein expression levels were detected among the treatment groups, indicating comparable anti-inflammatory outcomes.

Overall, these findings indicate that TCZ + DEX co-treatment alleviates airway inflammation, likely through reductions in NF-κB–associated inflammatory activity. Previous research has demonstrated that TNF-α activates the NF-κB signaling pathway and exacerbates airway inflammation [53]. Both TCZ and corticosteroids alleviate acute lung injury and diminish airway inflammation by modulating the NF-κB signaling pathway [54, 55]. Consistent with these findings, our research revealed that the combination of TCZ and DEX can alleviate inflammatory signals and lung tissue damage by reducing NF-κB activation. Although the effects of TCZ, DEX, and TCZ + DEX were comparable, it is noteworthy that the DEX dosage in the TCZ + DEX group was only half of that in the DEX group. These results suggests that combination therapy can maintain efficacy comparable to monotherapy while potentially reducing side effects, highlighting its promise as a safer and more effective approach for asthma management.

## Discussion

In this study, we systematically investigated the therapeutic potential and significant advantages of the TCZ and DEX combination strategy for asthma management through both in vitro and in vivo experiments. This work aimed to explore whether combining TCZ with a reduced dose of DEX could enhance anti-inflammatory efficacy while reducing GC-associated side effects.

In cell experiments, the combined treatment of TCZ and DEX integrates the anti-inflammatory effects of TCZ with the anti-inflammatory and antioxidant effects of DEX. This approach significantly improves cell survival rates by reducing the release of inflammatory mediators, alleviating oxidative damage, and promoting cell proliferation. To simulate airway epithelial injury in asthma, H_2_O_2_ was used to induce oxidative stress in BEAS-2B cells, effectively reproducing the inflammatory microenvironment of asthmatic airways. The results demonstrated that TCZ + DEX combination therapy markedly alleviated H_2_O_2_-induced oxidative damage and mitochondrial dysfunction while restoring cell viability and proliferation. These findings provide new insights into the protective role of TCZ + DEX against oxidative stress–related airway injury and suggest that this combination may serve as an effective strategy for diseases involving epithelial oxidative damage, such as asthma and COPD. Moreover, because TCZ acts through immunomodulatory and anti-inflammatory pathways independent of direct IL-6R inhibition in this human cell model, its combination with DEX may contribute to protective effects while enabling the use of a lower GC dose. In the animal experiments, the study evaluated the therapeutic effects of TCZ + DEX combination therapy in an asthma mouse model. It systematically assessed its impact on clinical symptoms, lung function, inflammatory response, and molecular mechanisms. The results show that TCZ + DEX significantly improves clinical symptoms and lung function in asthma mice. It also effectively alleviates airway inflammation and reduces inflammatory cytokine levels, primarily through suppression of NF-κB–associated inflammatory signaling, thereby attenuating the release of pro-inflammatory mediators. Given that TCZ exhibits limited affinity for murine IL-6R, these effects are more likely to reflect its indirect anti-inflammatory and immunomodulatory actions rather than direct IL-6R blockade. In addition, the combination therapy effectively reduced airway remodeling, suggesting that the addition of TCZ may help maintain therapeutic effects when using a lower steroid dose, which is consistent with the concept of reducing GC exposure when possible. This suggests that adjusting drug dosage in combination therapy may allow maintenance of therapeutic efficacy while reducing overall GC exposure, which could be relevant for clinical dose-sparing strategies. These findings further support the efficacy of combination therapy in reducing airway inflammation.

Beyond these mechanistic and functional findings, it is also essential to contextualize TCZ + DEX within the landscape of currently available biologic therapies. Compared with currently approved biologics for Type 2 asthma, such as omalizumab (anti-IgE), mepolizumab and benralizumab (anti-IL-5/IL-5R), and dupilumab (anti-IL-4Rα), TCZ exerted its broader immunomodulatory effects by regulating key inflammatory pathways. This difference may be clinically relevant for patients with mixed or non–Type 2 inflammatory phenotypes, or for those who respond inadequately to Type 2-directed biologics. Furthermore, both in vitro and in vivo findings consistently demonstrated that TCZ + DEX combination achieved anti-inflammatory and antioxidative effects. By concurrently suppressing inflammatory signaling and promoting epithelial repair, TCZ + DEX helps preserve airway epithelial integrity and suggests potential clinical benefits for chronic inflammatory airway diseases.

To further elucidate the molecular basis of these combined effects, future studies integrating transcriptomic or metabolomic analyses are warranted to elucidate the molecular targets through which TCZ and DEX jointly regulate mitochondrial homeostasis, which may further optimize therapeutic strategies against oxidative stress–related injury. Prolonged exposure to GCs, particularly at high doses, is known to contribute to undesirable effects such as impaired immune function and metabolic dysregulation [7, 56]. Therefore, TCZ combined with low-dose DEX could represent a complementary therapeutic approach that may help reduce overall GC exposure. In this study, the combined treatment achieved comparable anti-inflammatory effects with potential additive benefits, while potentially helping to reduce overall GC exposure, aligning with recent research trends emphasizing optimized combination therapy for asthma management [57].

Nevertheless, several limitations exist. First, some experiments involved relatively small sample numbers, which may have reduced statistical power and led to some group differences not reaching statistical significance. Future studies should increase the sample size to enhance reliability. Secondly, although this study explored NF-κB signaling as a key pathway, the specific molecular mechanisms by which TCZ modulates asthma remain to be clarified. Given that TCZ does not directly bind to murine IL-6R, its in vivo effects may reflect regulation of inflammatory and immune processes. Future work should investigate whether TCZ engages alternative or downstream IL-6–related signaling pathways that contribute to its immunomodulatory effects. Thirdly, while BEAS-2B cells provided mechanistic insights, they cannot fully replicate the complex airway environment. Future studies using primary airway epithelial cells from mice or asthmatic patients will help validate and extend these findings. Moreover, although our study provides insights into the anti-inflammatory effects of TCZ + DEX in vitro, cell cycle-associated factors were not assessed in vivo due to limited tissue availability and the prioritization of inflammation-related analyses. Future studies should evaluate key cell cycle regulators in lung tissues to elucidate the proliferative and regenerative effects of TCZ + DEX on the airway epithelium. Although TCZ + DEX combination therapy was associated with suppression of NF-κB signaling, future research should also clarify whether IL-6–related downstream pathways participate in these combined therapeutic effects. Additionally, while the combination therapy reduces the dosage of GCs, the long-term biological effects of this dose adjustment remain to be clarified, the immune-modulatory risks associated with long-term TCZ use have not been fully evaluated. Therefore, subsequent studies should refine dosing schedules and combination therapy strategies to ensure their long-term safety and clinical feasibility, ultimately advancing the translational potential of TCZ + DEX as a novel therapeutic approach for asthma.

## Conclusion

This study employed both in vitro and in vivo experiments to systematically evaluate the therapeutic efficacy of TCZ + DEX combination therapy for asthma and elucidate its underlying mechanisms. In vitro experiments, TCZ + DEX significantly alleviated H_2_O_2_-induced oxidative stress damage, modulated mitochondrial dysfunction, and inhibited apoptosis in BEAS-2B cells, demonstrating notable protective effects. In vivo experiments, TCZ + DEX effectively improved lung function in OVA-induced asthmatic mice, reduced inflammatory cell infiltration, suppressed airway remodeling, and was associated with decreased activation of NF-κB–related inflammatory signaling. This study provides preclinical evidence supporting the potential therapeutic efficacy of TCZ + DEX combination therapy for asthma, which may help reduce the need for higher doses of GCs and contribute to optimizing treatment strategies for chronic inflammatory diseases. Future studies should validate the long-term safety of this combination regimen and explore its applicability across diverse asthma phenotypes and patient populations to advance precision asthma treatment.

## Supplementary Information

Supplementary Material 1.

Supplementary Material 2.

## Data Availability

The data supporting the findings of this study are available from the corresponding author upon reasonable request.
