# Supplementary figures and images for: Modulation of the NF-κB signaling pathway by the combined strategy of tocilizumab and dexamethasone for asthma therapy
Source: Respir Res. 2026 Jan 8;27:44. doi: 10.1186/s12931-025-03458-5 (PMC12874903; doi:10.1186/s12931-025-03458-5)

Figure 7F

pp65

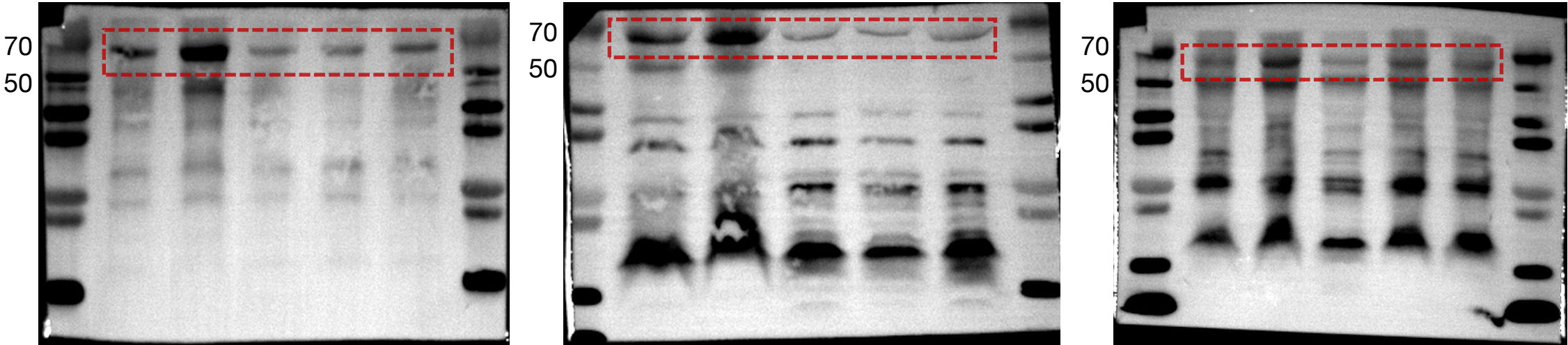

p65

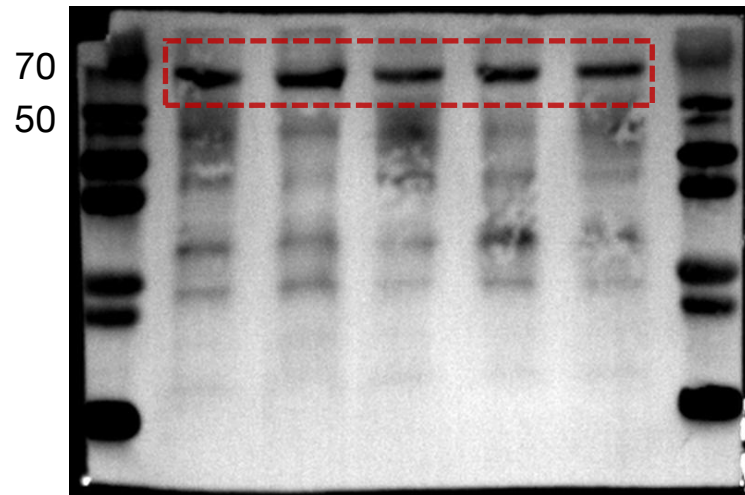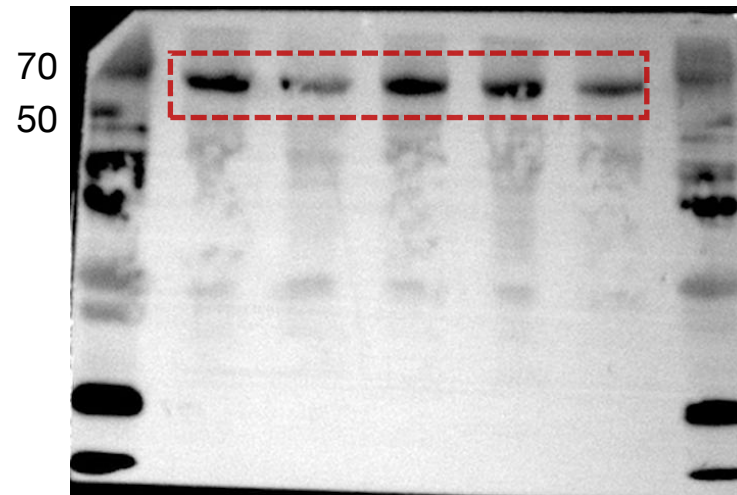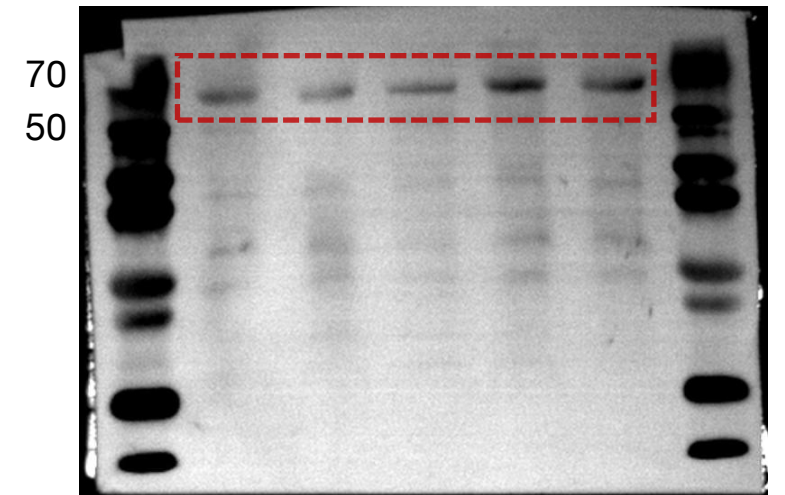

# GAPDH

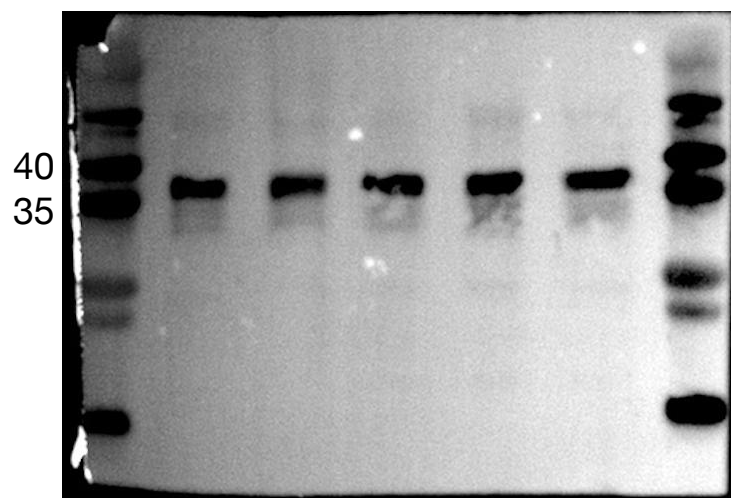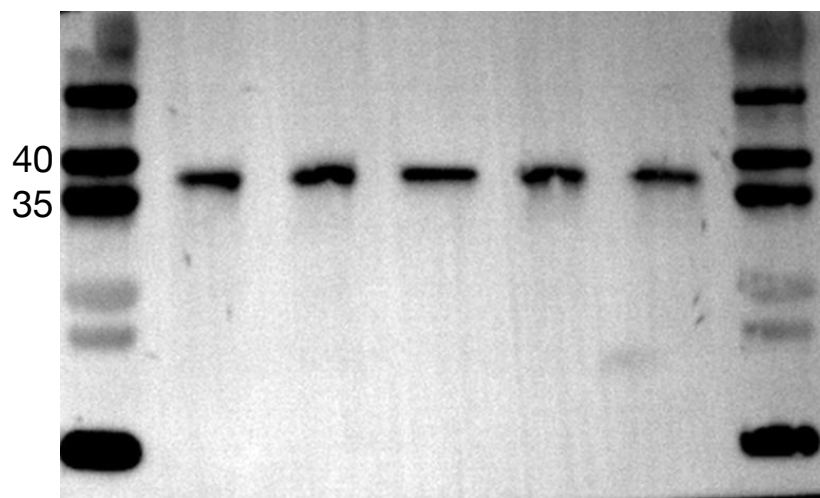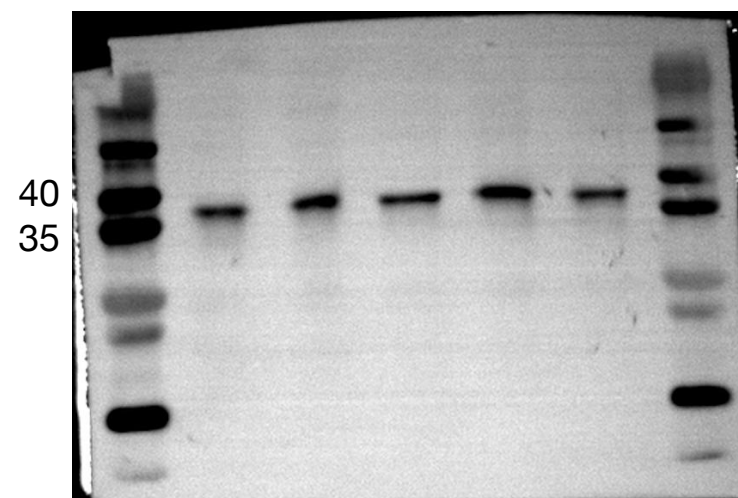

**TNF- $\alpha$**

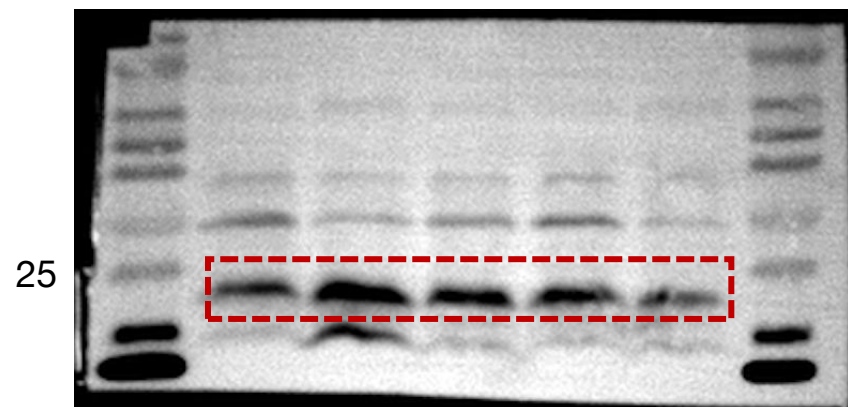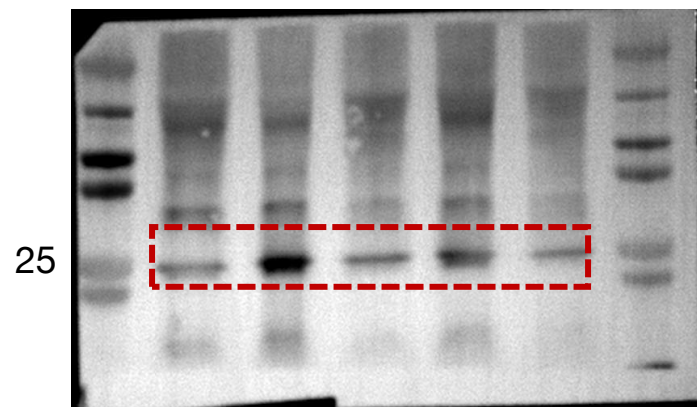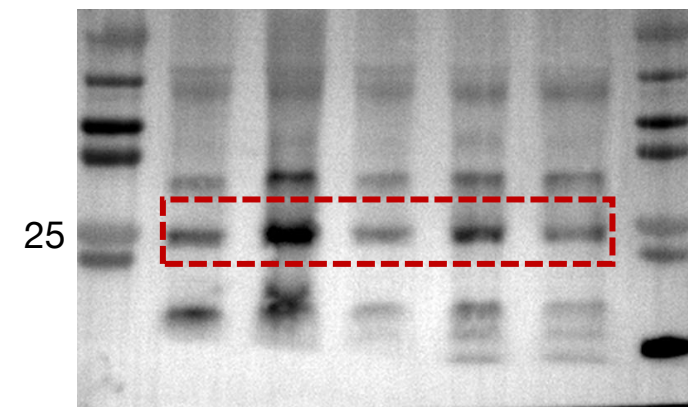

**$\beta$ -tubulin**

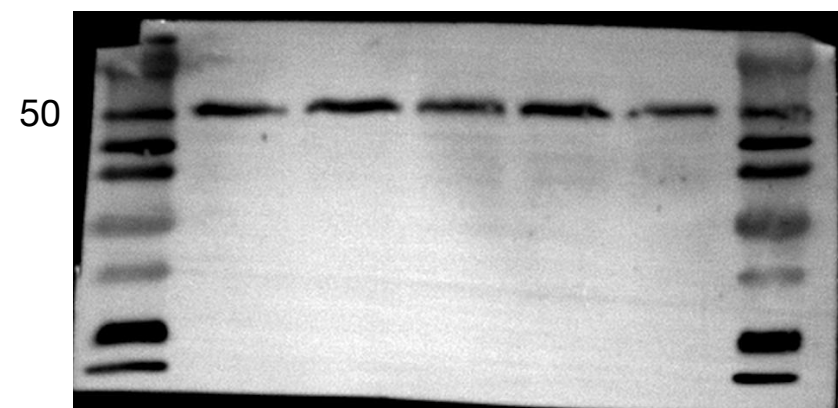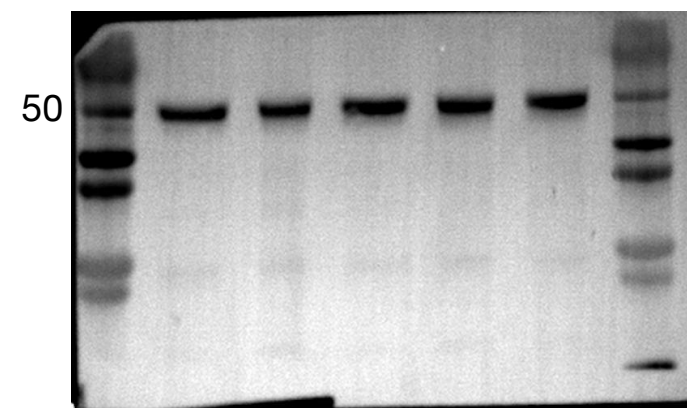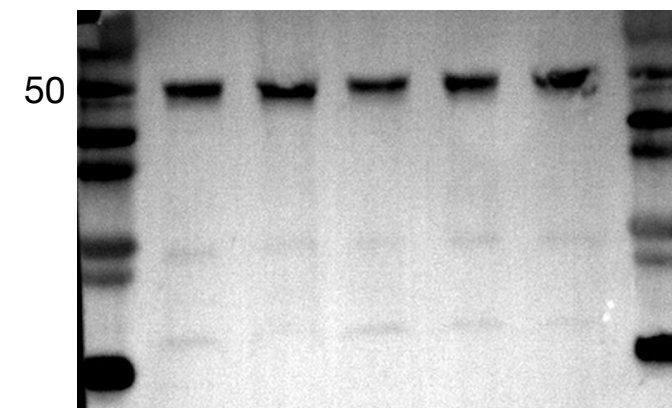

Supplement: Supplementary file 1 — Supplementary Material 1. [file 12931_2025_3458_MOESM1_ESM.pdf]
