# Supplementary material for: Modulation of the NF-κB signaling pathway by the combined strategy of tocilizumab and dexamethasone for asthma therapy
Source: Respir Res. 2026 Jan 8;27:44. doi: 10.1186/s12931-025-03458-5 (PMC12874903; doi:10.1186/s12931-025-03458-5)
Supplement: Supplementary file 2 — Supplementary Material 2. [file 12931_2025_3458_MOESM2_ESM.docx]

**Table S1**. Human gene primer sequences

| Gene | Forward (5′–3′) | Reverse (5′–3′) |
| --- | --- | --- |
| *IL6* | TGGCTGAAAAAGATGGATGCT | TCTGCACAGCTCTGGCTTGT |
| *TNF* | TGGCCCAGACCCTCACACTCAG | ACCCATCGGCTGGCACCACT |
| *RELA* | CCCACGAGCTTGTAGGAAAGG | GGATTCCCAGGTTCTGGAAAC |
| *BCL2* | ACGGTGGTGGAGGAGCTCTT | GCCGGTTCAGGTACTCAGTCAT |
| *BAX* | TGGCAGCTGACATGTTTTCTG | TCCCGGAGGAAGTCCAATG |
| *CASP3* | AGAAATTGTGGAATTGATGCGTG | ACAACGATCCCCTCTGAAAAAGT |
| *MKI67* | CAAAGAGTGAGAAAGGCAAAATCA | TGAACTTGCCGACTGCTAGGA |
| *CDK2* | GGACGGAGCTTGTTATCGCAAAT | CCTTGGCCGAAATCCGCTT |
| *CCND1* | GAGAAGTTGTGCATCTACACTG | AAATGAACTTCACATCTGTGGC |
| *CDKN1A* | AGCAGAGGAAGACCATGTGGA | AATCTGTCATGCTGGTCTGCC |
| *GAPDH* | CATGTTCGTCATGGGTGTGAA | GGCATGGACTGTGGTCATGAG |

**Table S2**. Mouse gene primer sequences

| Gene | Forward (5′–3′) | Reverse (5′–3′) |
| --- | --- | --- |
| *Il4* | TCACTGACGGCACAGAGCTA | CCTTCTCCTGTGACCTCGTT |
| *Il5* | GGCTGGCCTCAAACTGGTAA | CCCTGATGCAACGAAGAGGA |
| *Il13* | GGGATACCCACCGTTTAACCA | AGGTTTACTCTCCGAAAGCTCTT |
| *Tnf* | TGGCCTCCCTCTCATCAGTT | ATCGGCTGGCACCACTAGTT |
| *Rela* | GGCCTCATCCACATGAACTT | CACTGTCACCTGGAAGCAGA |
| *Actb* | GGCTGTATTCCCCTCCATCG | CCAGTTGGTAACAATGCCATGT |
